# Personalized Medicine for Classical Anesthesia Drugs and Cancer Progression

**DOI:** 10.3390/jpm12111846

**Published:** 2022-11-05

**Authors:** Bárbara Costa, Joana Mourão, Nuno Vale

**Affiliations:** 1 OncoPharma Research Group, Center for Health Technology and Services Research (CINTESIS), Rua Doutor Plácido da Costa, 4200-450 Porto, Portugal; 2 Centro de Investigação em Tecnologias e Serviços de Saúde (CINTESIS)@Health Research Network (RISE), Faculty of Medicine, University of Porto, Alameda Professor Hernâni Monteiro, 4200-319 Porto, Portugal; 3 Department of Anesthesiology, Centro Hospitalar Universitário de São João, Alameda Professor Hernâni Monteiro, 4200-319 Porto, Portugal; 4 Department of Community Medicine, Information and Health Decision Sciences (MEDCIDS), Faculty of Medicine, University of Porto, Rua Doutor Plácido da Costa, 4200-450 Porto, Portugal

**Keywords:** anesthesia, cancer recurrence, metastasis, drug interactions, pharmacokinetic, pharmacodynamic, in silico studies

## Abstract

In this review, we aim to discuss the use and effect of five different drugs used in the induction of anesthesia in cancer patients. Propofol, fentanyl, rocuronium, sugammadex, and dexamethasone are commonly used to induce anesthesia and prevent pain during surgery. Currently, the mechanisms of these drugs to induce the state of anesthesia are not yet fully understood, despite their use being considered safe. An association between anesthetic agents and cancer progression has been determined; therefore, it is essential to recognize the effects of all agents during cancer treatment and to evaluate whether the treatment provided to the patients could be more precise. We also highlight the use of in silico tools to review drug interaction effects and safety, as well as the efficacy of the treatment used according to different subgroups of patients.

## 1. Introduction

An area of ongoing attention is the determination of how anesthetic medications affect long-term oncological outcomes following cancer surgery. Surgery for cancer frequently makes use of anesthetics. Events that occur during surgery, such as being subjected to general or local anesthetic, may have an impact on the progression of cancer. Cancer patients undergoing surgery incur the risk of cancer recurrence and metastasis due to the triggering of the body’s natural stress response, which begins with increasing pro-inflammatory release, neuroendocrine signaling, and immunomodulation. However, depending on the type of cancer and how the therapy is administered, anesthesia may either inhibit cancer or serve as a catalyst for metastasis, which results in a detrimental effect on cancer growth [1,2].

**Figure 1:**
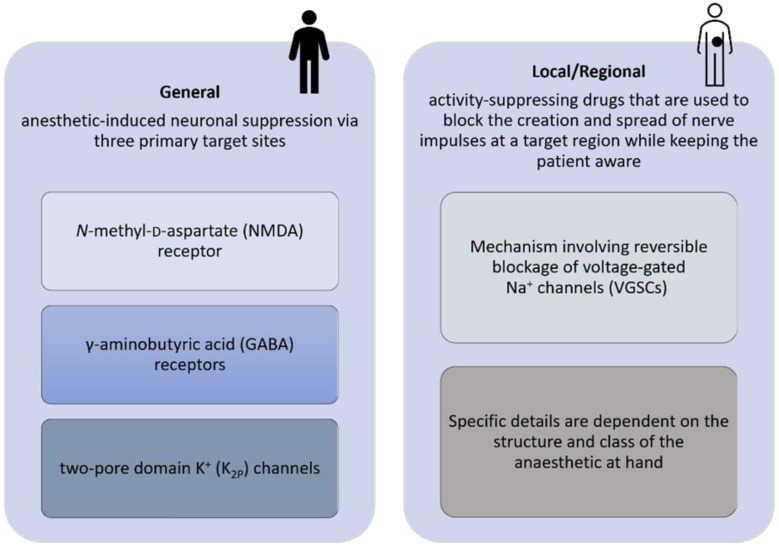
Diagram proposed for the pharmacology of general, local, and regional anesthetics.

Recent experimental results revealed that several of the anesthetics most frequently used in surgical oncology, whether general or local agents, can modify gene expression and epigenetic alterations [3,4]. Considering this, more studies should be developed to identify the best procedure (general or local/regional anesthesia) for any given patient population or disease. The use of different agents in anesthesia should be better understood to mitigate pro-inflammatory responses and regulate oncogenes, since different types of anesthesia assert different mechanism at play, see Figure 1.

## 2. Propofol and Fentanyl

**Figure 2:**
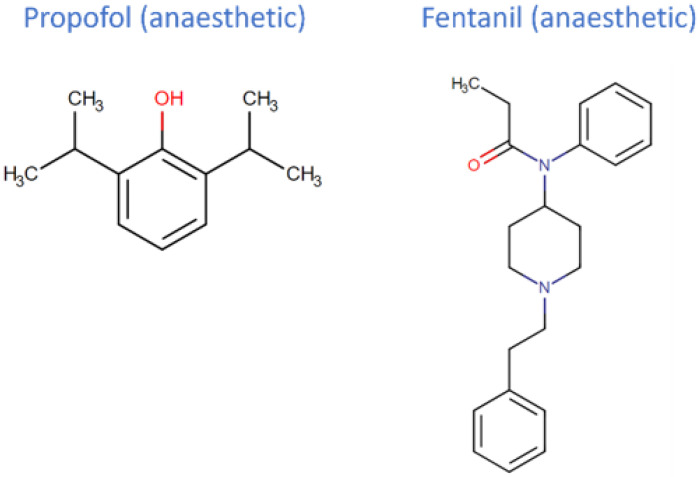
Chemical structure of the drugs propofol and fentanyl.

Modern medicine as we know it today would be inconceivable without the use of anesthetics. Using drugs to induce sedation and prevent pain during surgery is a game changer. The constant development in this field has allowed for faster induction times, quicker recovery, and a better understanding of the anesthetic’s mode of action. Anesthetics are classified as general, regional, and local. General anesthesia induces a reversible state of unconsciousness, amnesia, and analgesia. Local anesthesia numbs a small area of tissue for a minor procedure, for example, a tooth extraction. Regional anesthesia numbs a larger part of the body; however, the area is limited and the anesthesia does not render the patient unconscious. The pharmacology of the agents used differs according to the anesthetic category (Figure 2) [5].

No other medicine has had the same impact on anesthesia practice as propofol, which was introduced two decades ago. Propofol (2,6-diisopropylphenol) is an intravenous drug used for the induction and maintenance of sedation and general anesthesia. It acts as a GABA agonist, exhibiting rapid induction and a short half-life [6]. Propofol also depresses neuronal activity by inhibiting glycine, nicotinic, and the M1 muscarinic receptors [7]. The most prevalent side effect of propofol is pain upon injection, which is well-documented. The primary location for propofol metabolism is the liver (which is very efficient; therefore, it is necessary to maintain hepatic perfusion to maintain the propofol metabolic rate). Propofol is predominately glucuronidated by human UGT1A9 and intestinal or liver microsomes. Additionally, it can be in part metabolized by CYP 3A4, mostly by CYP 2B6 and CYP 2C9 [8,9].

Fentanyl is a narcotic analgesic, broadly used for analgesia, sedation, and anesthesia, in both adult and pediatric patients. It is a powerful opioid with high lipophilicity. Its use was restricted to anesthesia until the 1990s, when formulations of non-injectable fentanyl were developed, and its prescription as an analgesic was allowed. Fentanyl is involved in first-pass metabolism through CYP 3A4. Norfentanyl is the major metabolite produced from fentanyl; however, it is not known to produce clinically relevant pharmacodynamic effects [10].

Although propofol is commonly used, it leads to a sequence of undesirable hemodynamic effects (bradycardia and negative inotropism, among others). Due to a diminished central compartment and a lower concentration of plasma proteins, which result in a decreased distribution volume and cardiac output, older patients are more sensitive to propofol. The combination of propofol with other opioids, such as fentanyl, creates a synergistic effect [11]. The concentration of propofol required to induce unconsciousness and suppress responses to unpleasant stimuli can be much lower in the presence of fentanyl [12]. As post-intubation hypertension raises the risk of myocardial infarction, heart failure, pulmonary edema, cerebral hemorrhage, etc., concurrent fentanyl administration during anesthetic induction seeks to minimize the catecholaminergic response to laryngoscopy.

Due to pharmacological synergy, it has been suggested that opioids be administered first to reduce the requirement for propofol. Given the dose-dependent nature of propofol-associated hypotension, it has been hypothesized that a dose decrease based on opioid coadministration would lead to reduced hemodynamic instability [13]. Low-dose fentanyl coupled with propofol is safer, has a greater anesthesia effect, can lower the frequency of side effects in patients, and has some additional clinical benefits [14].

## 3. Complementary Drugs to Propofol and Fentanyl

**Figure 3:**
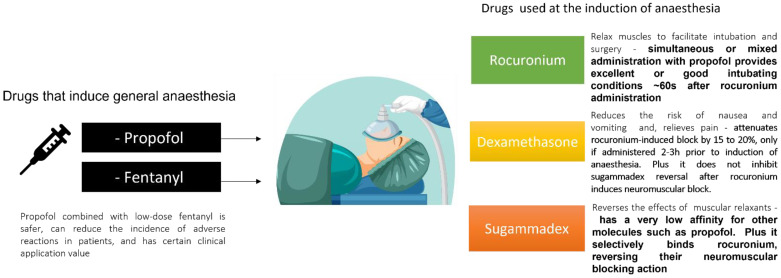
Correlation between the five drugs used during the induction of anesthesia.

**Figure 4:**
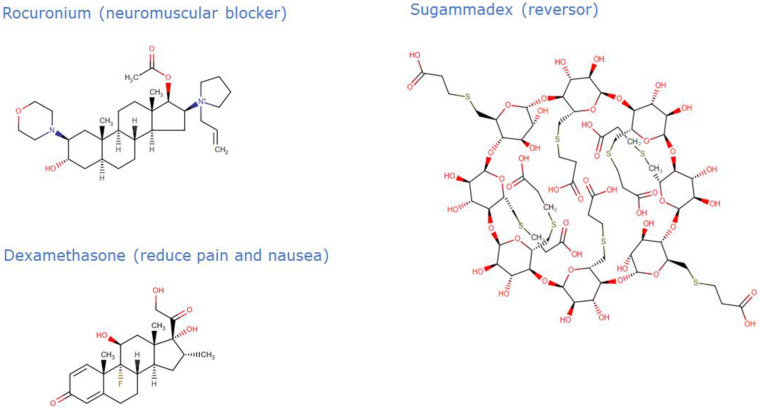
Chemical structure of the drugs rocuronium, sugammadex, and dexamethasone.

Numerous methods have been investigated to lengthen the effectiveness and boost the duration of local anesthetic nerve blocks. Some of these entail adding adjuncts or combining local anesthetics (Figure 3 and Figure 4). In this section, we aim to review the effects of the mixed administration of the drugs reviewed within intubation conditions to access two factors: (1) the improvements regarding, or the need for, the two combined together; and (2) the lack of research and information in this area.

Rocuronium is a non-depolarizing neuromuscular blocker (NMB) frequently used with general anesthesia to relax muscles to facilitate intubation and surgery in both elective and emergency scenarios. It has a characteristic advantage of being fast-acting and reversible when compared with other available non-depolarizing neuromuscular blocking agents in clinical anesthesia. It functions by competing for cholinergic receptors at the motor endplate. When it is time to counteract this activity, acetylcholinesterase inhibitors, such as neostigmine and edrophonium, can be used; however, sugammadex is the best antidote (it is efficient, even when given during profound blockage, and the coadministration of an anticholinergic agent is not necessary, in contrast to the characteristics of acetylcholinesterase inhibitors). Rocuronium works by binding to nicotinic cholinergic receptors in a competitive manner [15]. Moreover, it is metabolized to 17-desacetyl-rocuronium, a less active metabolite, and is eliminated primarily by the liver [16].

The synchronized or mixed administration of rocuronium with propofol allows for excellent intubating conditions, reducing the pain of propofol administration. Therefore, the timing of administration plays an important role. Rocuronium injection should occur after induction with propofol. Besides, low dose rocuronium improves conditions for tracheal intubation after induction of anesthesia with propofol [17].

Moreover, the combination of fentanyl with rocuronium has also been proven beneficial for intubation conditions. The association of opioids, such as fentanyl, with rocuronium can attenuate pain during administrations, fentanyl being more effective than lidocaine [18] and less effective than ketamine [19].

Sugammadex is a modified gamma cyclodextrin, and it is the first selective relaxant binding agent indicated to reverse the neuromuscular blockade (for example, induced by rocuronium) during general anesthesia used to facilitate surgical procedures [20]. Sugammadex has the potential to address many of the problems inherent in current anesthetic techniques related to rocuronium and other amino steroids muscle relaxants with antagonistic effects. Sugammadex is generally well-tolerated and free of adverse reactions. The discovery of sugammadex is, without a doubt, a significant development that will likely alter the course of clinical neuromuscular pharmacology, its cost being the main limitation of use. Sugammadex is quite close to being the perfect reversal agent, yet the ideal reversal agent has not yet been discovered [20,21]. Its mechanism of action works by encapsulating the molecules of neuromuscular blocking agents, facilitating the dissociation of their targets. Sugammadex is primarily excreted quickly and unaltered by the kidneys. No metabolites of sugammadex were observed during clinical studies [22]. Additionally, sugammadex can effectively reverse the neuromuscular block caused by rocuronium while allowing propofol maintenance anesthesia. Compared to other anesthetics sugammadex’s safety profile improves when combined with propofol [23]. Its unique mechanism allows a rapid and efficient reversal of the deep neuromuscular blockade, with no negative impact on autonomic cholinergic systems [24].

Dexamethasone is a glucocorticoid commonly used in anesthesia and surgery to reduce the risk of nausea and vomiting, relieve pain, and make the patient feel better. However, there are different indications for different formulations of dexamethasone. Dexamethasone’s mechanism of action is unclear; however, it is most likely the result of both non-genomic and genomic anti-inflammatory activities [25]. The use of perioperative dexamethasone has been clinically proven to be beneficial. Many people believe that dexamethasone is the ideal peri-operative medication because it is affordable, easily accessible, encourages appetite and a sense of well-being, and is linked to a quicker discharge from day surgery; therefore, it is frequently utilized. Nonetheless, some patients can suffer from side effects associated with long-term steroid use [26]. Overall, dexamethasone’s side effects are uncommon, and its advantages outweigh the risks.

After some surgeries (e.g., tonsillectomy) postoperative vomiting is very common; according to Erdem et al., intraoperative subhypnotic propofol infusion, in combination with dexamethasone, provides a better postoperative vomiting prophylaxis than dexamethasone alone, which is a practice that is beneficial to the patient [27]. However, if pretreatment with dexamethasone is provided before propofol, patients develop perineal pruritus that can cause extreme discomfort.

Dexamethasone and fentanyl combined significantly attenuated the ACTH response after surgery, even though dexamethasone and fentanyl taken separately had no impact. Glucocorticoids and morphine agonists have mutually inhibiting effects on the production of ACTH in humans, most likely because they reduce the release of CRH from the hypothalamus [28]. A different study reported that pre-treatment with a bolus injection of dexamethasone prior to fentanyl administration reduces fentanyl-induced cough [29].

Moreover, sugammadex encapsulates the cyclopentanoperhydrophenanthrene ring of rocuronium, inactivating it, which results in the reversal of the neuromuscular block. Rocuronium and dexamethasone both have steroidal structural similarities. Buonanno et al. concluded that dexamethasone does not interfere with the reversal of neuromuscular blockade, although some in vitro studies suggest an impairment [30]. Recently, a meta-analysis on the effect of dexamethasone on the sugammadex reversal of rocuronium-induced neuromuscular block confirms the results discussed above. No effect was observed regarding the time required to extubate patients following general anesthesia or the time required for the neuromuscular reversal of sugammadex [31,32]. If taken 2/3 h before inducing anesthesia, a single dose of dexamethasone 8 mg reduced the rocuronium-induced block by 15–20%. In addition, dexamethasone administered during the induction of anesthesia had no effect on how long the neuromuscular block lasts [33].

## 4. Effect on Cancer Treatment

**Figure 5:**
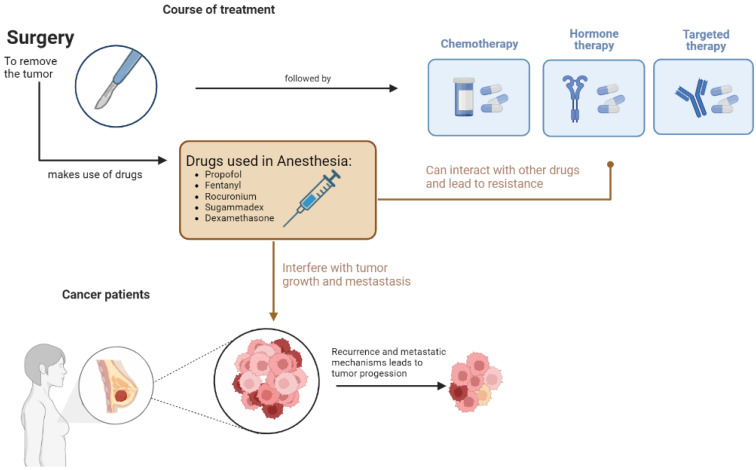
Schematization of the effect of drugs used in anesthesia in cancer progression and metastasis.

Agents used to maintain and induce general anesthesia have anti-inflammatory and immunomodulatory effects [34]. According to the literature, regional anesthetics can protect cell-mediated immunity, reduce neuroendocrine stress, and may reduce the need for opioids [35]. On the other hand, local anesthetics can block the VGSC activity during or after surgery, resulting in a reduced ability of the cancer cells to escape from the preoperative area, and can thus increase chemo-sensitization, Figure 5 [36].

Propofol is a widely used anesthetic that can be correlated with anti-tumor and cancer-inducing effects by regulating different signaling pathways. A few studies have shown that propofol stimulated nuclear factor E2 and related factor 2 (Nrf2) at the transcriptional and translational levels, promoting cell proliferation and invasion in gallbladder cancer in a dose- and the time-dependent manner [37]. Accordingly, in human breast cancer MDA-MB-231 cells, propofol increased cell migration via activating the Nrf2 signaling system and stimulating cell proliferation, in part by downregulating the expression of p53 [38]. However, numerous studies have produced the opposite findings for different cancers. More recently, this dysfunction of metabolic reprogramming was documented on gastric cancer cells; however, in this case, the cell proliferation was suppressed through the Nrf2-mediated polyol pathway [39].

Furthermore, propofol in combination with chemotherapeutic drugs alters the effectiveness of chemotherapeutic treatment. For instance, through the EGFR/JAK2/STAT3 pathway, propofol increased the chemosensitivity of cervical cancer cells to cisplatin-induced death [40]. Another example is the result that treatment with propofol and cisplatin increased the FOXO1 pathway’s activity in ovarian cancer cells, promoting cell death [41]. Another study discovered that propofol increased the chemosensitivity of pancreatic cancer cells to gemcitabine by decreasing the activation of the NF-B signaling pathway induced by gemcitabine. Moreover, propofol can epigenetically regulate trastuzumab resistance in breast cancer [42]. Interestingly, in numerous clinical studies, it was discovered that propofol has no impact on the prognosis of various malignancies; according to several retrospective studies, propofol did not affect the prognosis for other cancer types after surgery [43]. In a retrospective cohort analysis, for instance, there was no difference in mortality or the rate of locoregional recurrence at 5 years following propofol therapy for breast cancer surgery [44]. These results were in accordance with those of earlier studies. A retrospective study of NSCLC patients found no difference in overall survival (OS) or relapse-free survival (RFS) between the propofol group and the inhalation group [45]. In a recent trial, the use of propofol intraoperative anesthesia had no effect on OS or progression-free survival in patients with high-grade gliomas undergoing cancer surgery (PFS) [43]. Similarly, there was no difference in cancer-related mortality among patients who underwent surgery for stomach cancer, whether or not propofol was used.

Although numerous in vitro and in vivo studies point towards a beneficial use of propofol for certain cancers, clinical retrospective and prospective studies assessing the connection between propofol and prognosis have thus far present contradicting conclusions: either propofol has no influence on survival outcomes, or it can increase survival in many cancer patients. For example, propofol treatment for colon cancer resulted in better OS and disease-free survival (DFS) and reduced postoperative metastases [46]. Nonetheless, these findings have some important limitations. Interestingly, many investigations have revealed that the initial site of cancer may have an impact on the various effects of propofol on oncogenic outcomes. Moreover, most of the research continues to demonstrate that propofol improves prognosis. Cancers that seem to have a better prognosis after utilizing propofol during the surgery encourage its use in favor of the patient. Prospective multicenter studies, with bigger sample sizes, are currently being conducted for malignancies in which propofol did not affect the prognosis [43]. To better understand the impact that anesthesia has on the development of cancer and to influence the selection of anesthetics used during cancer surgery, additional animal experiments and prospective clinical studies are required.

Opioids are powerful immunomodulators affecting NK cells activity and cytokine production, being pro-angiogenic through the activation of VEGF-receptors [47]. Fentanyl can have an effect on NK-cell activity and lymphocyte proliferation [48]. Moreover, the use of opioids for the treatment of pain in cancer patients is very common post-operatively. Fentanyl suppresses the growth and invasion of lung cancer by upregulating miR-331-3p and suppressing HDAC5 [49]. The results of the study demonstrated that fentanyl treatment reduced the number of cancer cells and cancer stem cells in the PANC-1 cell population, decreased the expression of stem cell markers, and increased the expression of genes related to apoptosis. These findings suggest that fentanyl, which is frequently used to relieve pancreatic cancer pain, may be an alternative for pancreatic cancer treatment.

The effect of fentanyl on chemotherapeutic drugs after cancer surgery has been well explored; however, no evidence emerges relating reduced sensitivity of cancer cells to chemotherapy. In a cell proliferation study, the effect of fentanyl and 5-fluorouracil was assessed in human colon cancer cell lines, and there was no evidence of affected sensitivity [50]. Moreover, in patients with ovarian cancer treated with anthracycline, fentanyl was considered safe for use [51]. However, strong CYP3A4 iso-enzyme inhibitors, such as itraconazole, can increase the plasma concentrations and pharmacologic effects of fentanyl, due to this CYP3A4 inhibitor’s potential reduction in the metabolic clearance of this opioid. More examples may follow, as bicalutamide and dexamethasone, which also affect the CYP3A4 enzyme, can increase or decrease (respectively) the level or effect of fentanyl [52]. Therefore, it is necessary to watch for the cumulative narcotic effects of fentanyl when this drug is administered with another chemotherapeutics.

Rocuronium can be correlated with the invasion, adhesion, and migration of cancer cells. Jiang et al. evaluated these effects on breast cancer cells (MDA-231) and gastric cancer cells (SGC7901, and BCG 823) [53,54]. Additionally, another in vitro study on MRC-5 cells with rocuronium demonstrated a reduced expression of stromal cell-derived factor-1 (SDF-1). This study indicates the effect of anesthetics on fibroblasts, a component of the tumor microenvironment [55]. These results alone only shed light on how cancer surgery must be optimized to lower the risk of recurrence and metastasis; however, more research is needed to confirm these results (in vitro, in silico, and clinical trials).

Sugammadex is considered to enhance recovery after surgery in cancer patients, and a link between its effect on chemotherapy is only documented for breast cancer patients. Sugammadex is related to growth hormone activities [56], also binding to steroid-structure molecules [57], such as estrogen and tamoxifen. Therefore, different agents should be given to patients on tamoxifen to reverse NMB. Otherwise, sugammadex may not be as effective in removing rocuronium [58]. The same applies for the drug toremifene, which is structurally similar to tamoxifen. We could not find any articles reporting the effect of these drugs on cancer cells, and this may be due to the size of this drug, as sugammadex does not enter the cells [59].

Dexamethasone is already used in combination with other drugs as a treatment for certain types of cancer, such as lymphoma, leukemia, or multiple myeloma, and it is widely used, either alone or with other drugs, to prevent certain conditions related to cancer (e.g., drug hypersensitivity). In multiple myeloma, dexamethasone in combination with lenalidomide improves the success of treatment by extending time-to-progression and survival. Dexamethasone reduces inflammation and suppresses the immune response by inhibiting CD28-mediated cell cycle entrance and differentiation, while upregulating CTLA-4 in the CD4 and CD8T cells. These data imply that corticosteroids hinder the response in immunotherapy. Giles et al. observed that with the injection of CTLA-4 for inhibition, T cells might be somewhat shielded or saved from the immunosuppressive effects of dexamethasone. Additionally, after an effective anti-tumor immune response, harmful corticosteroid effects are reduced. This research indicates that the effectiveness of immunotherapy is greatly influenced by the timing of dexamethasone treatment in relation to the emergence of anti-tumor immunity [60].

Moreover, before chemotherapy, dexamethasone is used in breast cancer patients to prevent adverse chemo effects. Several studies have shown that this pre-treatment improves the chemotherapy effects against breast cancer and may prevent metastasis in breast cancer by diminishing cell adhesion and migration. Mohammadi et al. observed a decrease in viability in T47D breast cancer cells. However, a different study stated that dexamethasone could promote the lung metastasis of breast cancer via the PI3K-SGK1-CTGF pathway, both in vitro and in vivo [61]. Dexamethasone pre-treatment prior to paclitaxel is a routine method used in the treatment of breast cancer, and metastasis caused by chemotherapy drugs has been documented in the past. Dexamethasone provides a stronger pro-metastatic ability [61]. These findings should prompt serious reservations about the therapeutic application of paclitaxel or other treatments in breast cancer patients. In chemotherapy, dexamethasone is still a commonly utilized pre-treatment drug due to its durable effects and generally affordable. Keep in mind that dexamethasone is still frequently used as a pre-treatment medication in the treatment of tumors because it successfully lessens the symptoms regarding nervous system compression and vomiting caused by chemotherapy, as well as prevents radiotherapy-related toxicity and other side effects.

Interestingly, in the MCF-7 and MDA-MB-231 xenograft mouse models, the administration of low-dose dexamethasone decreased tumor growth and distant metastasis, but treatment with high-dose dexamethasone increased tumor growth and metastasis, respectively. Breast cancer cells subjected to dexamethasone treatment showed a dose-dependent inhibition of cell adhesion, migration, and invasion. Part of the manner in which dexamethasone inhibits cell adhesion, migration, and invasion in MDA-MB-231 cells is via causing the induction of microRNA-708 and the subsequent mediation of Rap1B-signaling. On the other hand, the dexamethasone suppression of cell migration in MCF-7 cells does not depend on microRNA-708-mediated signaling. Overall, dexamethasone has a double role in the progression and metastasis of breast cancer: While larger amounts might unintentionally advance breast cancer, lower quantities prevent the growth and metastasis of breast cancer tumors [62], highlighting the importance of using in silico studies and further developing precision medicine to guarantee the proper treatment.

**Figure 6:**
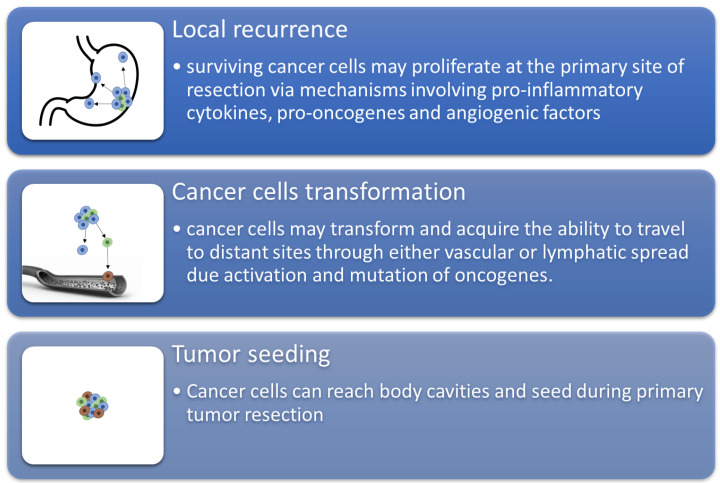
The schematization of recurrence and metastasis through three main mechanisms.

In conclusion, it is interesting to acknowledge that individuals could benefit or suffer, depending on the drugs used to induce and maintain general anesthesia during surgical intervention. There is inadequate evidence to support the use of certain anesthetic agents or techniques to lower the risk of cancer recurrence in individuals who undergo cancer surgery, despite laboratory, animal, and retrospective human data suggesting that anesthetic agents may alter cancer recurrence (Figure 6). The use of in silico studies is a tool that we can use in our favor to gain more information and make more powerful and informed decisions during treatment.

## 5. Potential Opportunities from In Silico Studies and Pharmacokinetics (Pk)/Pharmacodynamics (PD) Models

For the proper administration of anesthetics, it is essential to have a good grasp of pharmacokinetics and pharmacodynamic interactions (Table 1). Safe and effective combinations of numerous agents are required to deliver optimal anesthesia and anti-cancer treatment. To appropriately dose propofol administration during anesthesia, numerous pharmacokinetic models have been created. Commercial propofol target-controlled infusion (TCI) syringe pumps use pharmacokinetic models created by Marsh et al. and Schnider et al. [63]. Moreover, it would be useful to utilize the PK and PD of anesthetics in cancer procedures in different populations to understand which anesthetic is the most advantageous.

In 2015, a population PK/PD model of propofol was suggested for cancer patients undergoing major lung surgery. This investigation comprised 23 individuals who received complete intravenous anesthesia with propofol and fentanyl. The PK of propofol could be adequately described by a three-compartment model. A sigmoidal E max model was used to relate the anesthetic effect (AAI index) to the concentrations at the propofol effect sites. When comparing the values for healthy volunteers given by Schnider et al. and Eleveld et al. in the literature, it was found that the population in the study had somewhat higher clearance values, lower distribution clearance values, and a smaller volume of the peripheral compartment. Despite these variations, the median performance error of both models indicated a clinically negligible bias of between 8 and 1% in concentration estimations. Most investigated factors had no appreciable impact on propofol’s PK/PD [67].

A different study described the pharmacokinetics of 52 patients treated with subcutaneous and/or transdermal fentanyl for moderate to severe cancer-related pain. The results were well represented by a one-compartment model, with first-order elimination and distinct first-order absorption processes for each route. Variability across individuals and across occasions ranged from moderate to high. The measured and simulated plasma fentanyl concentrations increased, and increased adverse effects were seen from both subcutaneous and transdermal fentanyl [68]. A different study regarding the pharmacokinetics of intranasal fentanyl spray in patients with cancer was conducted to assess the tolerability and safety of this spray [69]. Neither of these studies measured the effect of the different formulations of fentanyl on cancer progression or the interaction with other drugs.

Recently, an in-silico study using Gaussian accelerated molecular dynamics allowed us to further understand the interactions between propofol and fentanyl. Multiple binding sites were identified, demonstrating that fentanyl acts as a stabilizer of propofol on binding sites [70]. More studies like this one are necessary to further extend our knowledge regarding the mechanism of action and the drug interaction of anesthetics.

Furthermore, no population PK/PD analysis for sugammadex or rocuronium was found regarding their effects on cancer patients and drug interactions. Regarding the interaction of rocuronium with cancer cells, this in silico study could be of great potential.

We did indeed more easily find a population PK study regarding dexamethasone in cancer patients; this study regarded the impact of the dose and duration of dexamethasone in pediatric acute lymphoblastic leukemia patients. There was significant interpatient pharmacokinetic diversity between the patients receiving various dosage levels, and from a clinical pharmacology standpoint, the length of dexamethasone therapy was demonstrated to be more significant than the precise dose [71]. Moreover, in 2020, a PK/PD model was created to quantify the pre-clinical anti-cancer effect of dexamethasone in pancreatic cancer, to facilitate future translational applications [72]. It is not surprising to find in silico studies modeling and simulating dexamethasone with cancer patients, since this drug has already been approved for certain types of cancer. However, we could not find drug interaction studies concerning dexamethasone and chemotherapeutics in cancer patients.

To administer anesthesia, a drug interaction is essential. For analyzing drug interactions, a response surface model (RSM) is a highly helpful tool. RSMs have been used to guide therapy to monitor the patient’s wellbeing, estimate wake-up times, forecast patient reaction, determine the ideal medication concentration, and support operations that call for rapid patient arousal. One model is not always superior to the others. Researchers are urged to use an objective metric to identify the model that best fits various situations [73].

Regarding the use of in silico studies, the development of a pharmacokinetic model to describe the complex pharmacokinetics of anesthetic interaction with chemotherapeutics would provide many benefits and improve cancer treatments and decisions, making a more tailored approach to patient treatment possible. More advanced forms of in silico experiments, using machine learning, quantitative pharmacological systems, and biological systems to understand the mechanistic pathways of drug–target interactions, absorption, trafficking, metabolism, side effects, and disease biology are required, using mathematics and statistics to solve biological problems.

## 6. Conclusions

Anesthetics play a role in cancer progression, and that role should be accounted for when deciding and planning the best cancer treatment for the patient. In this review, we sought to understand the role of five specific drugs used in anesthetics (these five drugs are commonly used in Hospital São João). After reviewing their mechanism of action and their potential role in the treatment of cancer, we believe that the use of in silico tools is fundamental to increase our knowledge regarding this matter. There is a current research gap in this specific area that should be explored to make our cancer treatments more precise. Personalizing treatment involves accounting for all the factors and variables. In our perspective, being aware of the anesthetics used and their interactions with other drugs is necessary for the success of these therapies.

## Figures and Tables

**Table 1:** The potential opportunities from in silico studies and Pk/PD models.

STANDARD MODELS	
Pk Models for Anesthesia Administration	Application
**Marsh et al.** [64]	Propofol target-controlled infusion (TCI) syringe pumps
**Schnider et al.** [65]	
**Eleveld et al.** [66]	Predicting propofol concentrations and the bispectral index
**Necessary studies to model Pk/PD of anaesthetics drugs for cancer patients**	
**Objective of the study:**	
1. Assess ADMET properties of all anesthetic drugs used in cancer patients (e.g., Gaussian accelerated molecular dynamics) to further understand the interactions between propofol and fentanyl;	
2. Assess the interaction of anesthetics drugs with the chemotherapeutics;	
3. Model tumor growth and assess the involvement of drug anesthesia in its progression and metastasis (e.g., multiparametric imaging data).	

## Data Availability

Not applicable.
